# Supplementary material for: New Fully Automated Preparation of High Apparent Molar Activity 68Ga-FAPI-46 on a Trasis AiO Platform
Source: Molecules. 2022 Jan 20;27(3):675. doi: 10.3390/molecules27030675 (PMC8840169; doi:10.3390/molecules27030675)
Supplement: Supplementary file 1 [file molecules-27-00675-s001.zip › molecules-1517102-supplementary.pdf]

**Supporting Information**

# New fully automated preparation of high apparent molar activity $^{68}$Ga-FAPI-46 on a Trasis AiO platform

Chiara Da Pieve[1], Marta Costa Braga[1], David R. Turton[1], Frank A. Valla[2], Pinar Cakmak[3,4,5], Karl-Heinz Plate[3,4,5], Gabriela Kramer-Marek[1]

[1] Preclinical Molecular Imaging, Division of Radiotherapy and Imaging, The Institute of Cancer Research,
[2] Sofie, Totowa, NJ, USA
[3] Institute of Neurology (Edinger Institute), University Hospital Frankfurt, Frankfurt, Germany
[4] German Consortium for Translational Cancer Research (DKTK), partner site Frankfurt/Mainz, Frankfurt, Germany
[5] Frankfurt Cancer Institute, Frankfurt, Germany

Contents

UV-Vis spectrum of FAPI-46 and structure of $^{68}$Ga-FAPI-46 page 2

RP-HPLC of FAPI-46 and $^{nat}$Ga-FAPI-46 page 3

RP-HPLC of product after SPE purification and stability page 4

ITLC analysis of product page 8

PET imaging page 9

Biodistribution table page 10

## UV-Vis spectrum of FAPI-46 and structure of $^{68}$Ga-FAPI-46

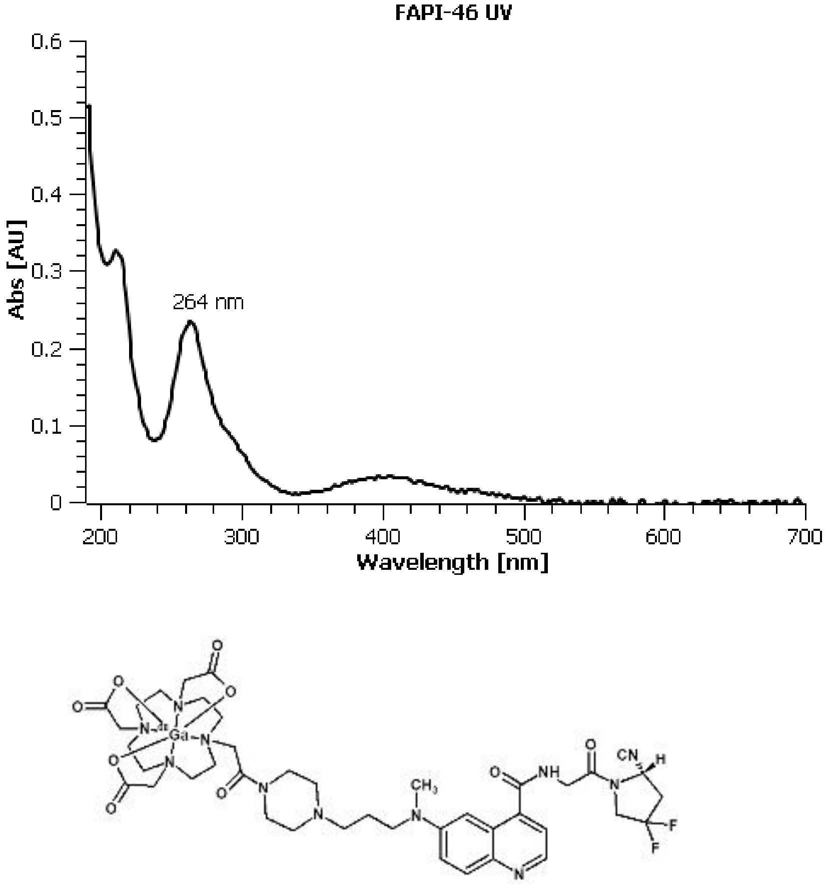


**Figure S1.** UV-Vis absorption spectrum of FAPI-46 and structure of $^{68}$Ga-FAPI-46.

## RP-HPLC of FAPI-46 and $^{nat}$Ga-FAPI-46

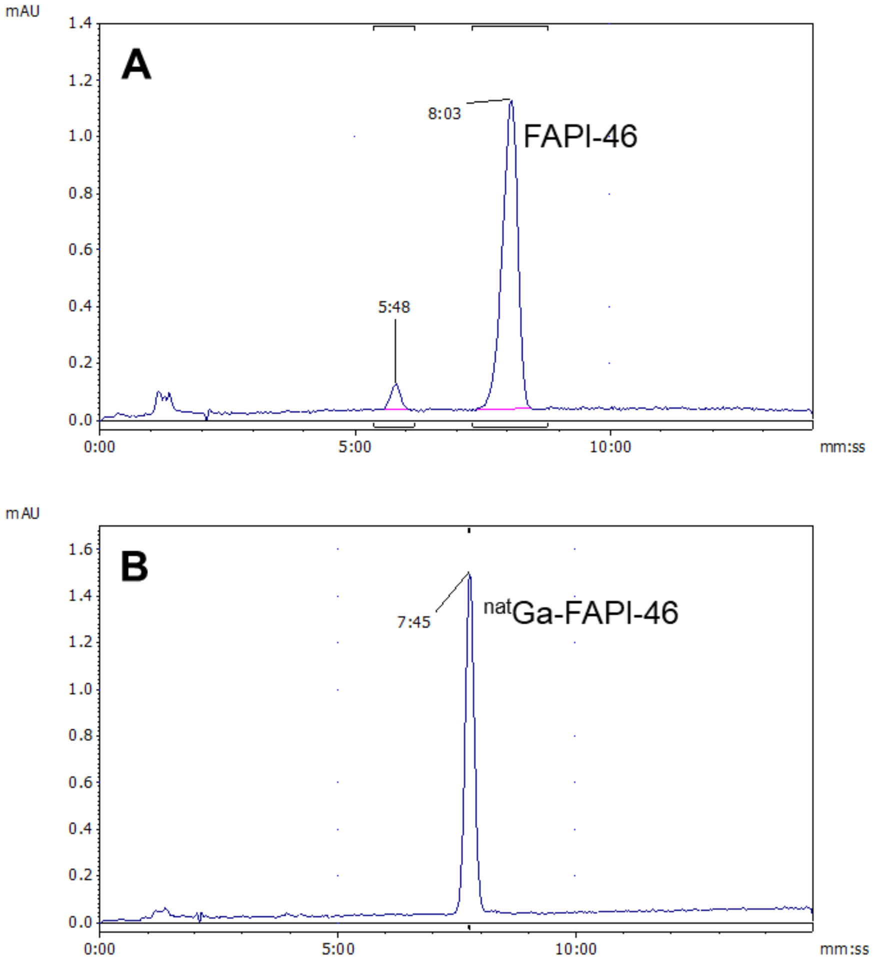


**Figure S2**. RP-HPLC analysis of FAPI-46 (**A**), and the $^{nat}$Ga-FAPI-46 (**B**). The absorbance was recorded at the wavelength of 260 nm. The retention time ($R_t$) is indicated as min:s.

## RP-HPLC of product after SPE purification and stability

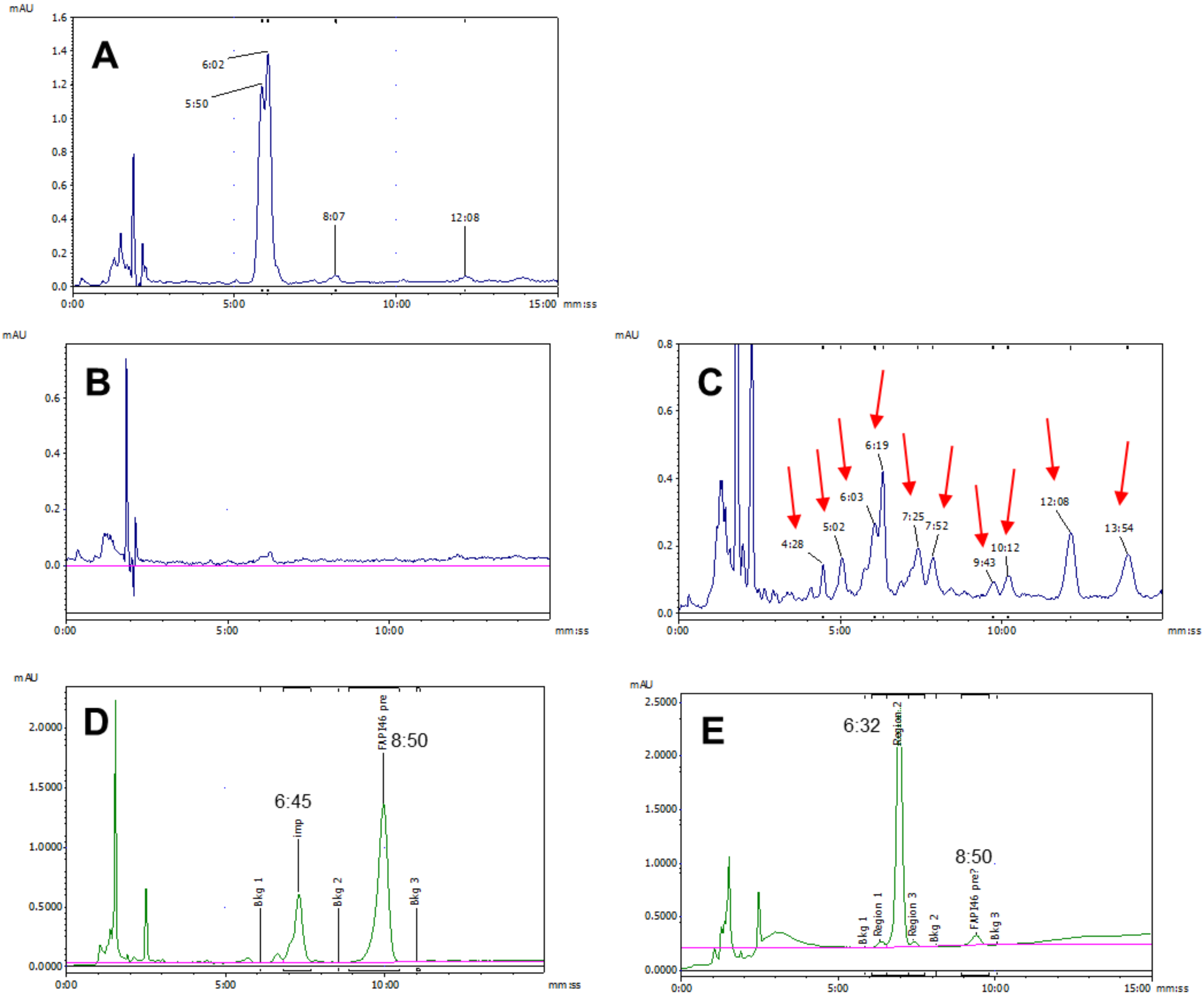


**Figure S3**. RP-HPLC analysis of $^{68}$Ga-FAPI-46 after purification by HLB cartridge only (1µL injection **A**) showing the presence of main unknown impurities eluting at 5:50-6:02 min:s. Those impurities are absent in the chromatograms of the product purified by combination of HLB + QMA cartridges (1µL injection **B**; 10µL injection **C**). The absorbance was recorded at the wavelength of 260 nm. The sum of the areas of the peaks indicated with a red arrow (in **C**) was used to calculate the apparent molar activity of the final product. The retention time ($R_t$) is indicated as min:s.

Molar activity (MA) values in the region of 2700-6390 MBq/nmol were calculated (by calibration curve) when only the area of the UV peak of the product (with a $R_t$ of approximately 7:45 min:s) was taken in consideration. However, limitations of the HPLC analytical method affected the appropriate extrapolation of MA values by introducing considerable errors (i.e. unreliable integration of the UV peak of the product due to a combination of poor resolution and product peak areas that were unpredictably under or very close to the limit of detection). Additionally, a variety of UV absorbing material was present in the final product solution. Assumed to derive from the precursor and therefore expected to bind competitively to FAP, this UV absorbing material was included in the MA calculations. The apparent molar activity (AMA) of the

product was thus determined and considered as a more accurate and reliable measurement than the MA.
The HPLC analysis shows the total disappearance of the precursor ($R_t$ = 8:03 min:s) and the presence in solution of a variety of compounds with different elution time from the product ($R_t$ = 7:45 min:s) (see Fig. S2). These compounds could result from the thermal decomposition of FAPI-46 during the radiolabelling reaction. To test this hypothesis, FAPI 46 in buffer at pH 4 was heated at 95°C in a HPLC vial and analyzed by HPLC. The chromatogram shows the presence of just two main compounds: the precursor (Rt = 8:50 min:s) and of an unidentified decomposition product (i.e. 27%) eluting at 6:48 min:s (after 20 minutes of heating) (**D**).
Some of these compounds could also be the complexes of FAPI-46 with non-radioactive metals (such as iron, nickel, or chromium) that might be present in traces in the solution. These metal contaminants could derive from the stainless steel needles and glass vials used in the production process and probably introduced in the reaction mixture after the purification of the $^{68}$Ga eluate by SCX. To explore this hypothesis, a 100 Sterican 0.98 × 70 mm (20G × 2¾”) needle was heated in NaCl/HCl mixture (chosen as lixiviant for the test as it is used in the synthetic procedure) for about ten minutes. The solution was then mixed with acetate buffer to have a final pH of 4-5, incubated at 95°C and analyzed by HPLC. The considerable disappearance of the precursor was detected already after 1.5 min of incubation together with the formation of one main product eluting at 6:32 min:s (**E**). Presumably, the needle releases traces of metals into the solution that can be complexed by the DOTA chelator in FAPI-46 (to form the product eluting at 6:32 min:s). However, additional studies (including HPLC analysis a different wavelengths) should be performed to fully identify the impurities in both the radiolabelling mixture and product solution and verify the previously mentioned speculations.
Importantly, as shown by the high RCYs and molar activities achieved during our experiments, the $^{68}$Ga-FAPI-46 formation was not seriously compromised by the presence of the above mentioned impurities independently from their nature.

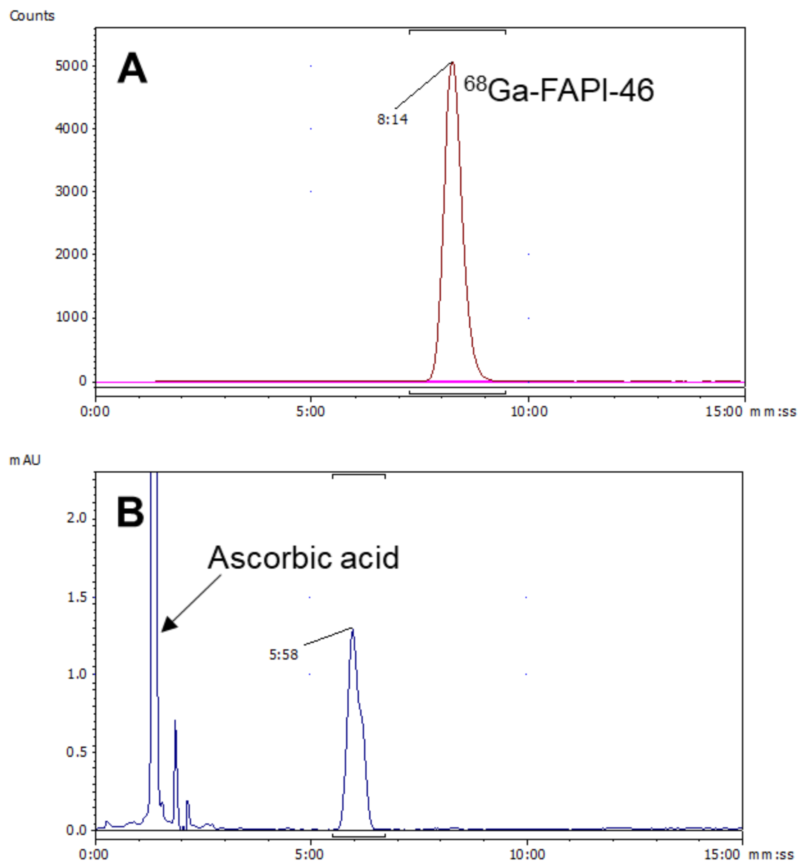


**Figure S4**. Radiochromatogram (RCP>98%) (**A**) and UV chromatogram (**B**) of $^{68}$Ga-FAPI-46 purified by QMA followed by HLB cartridge (1µL injection) showing the presence of an unknown impurity eluting at 5:58 min:s. The absorbance was recorded at the wavelength of 260 nm. The retention time ($R_t$) is indicated as min:s.

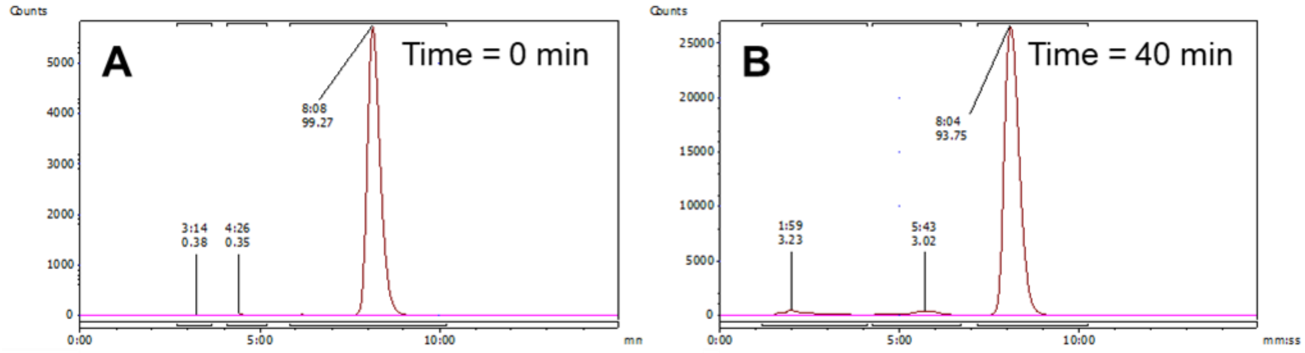


**Figure S5**. No ascorbic acid added to the final product: the radiochromatogram of $^{68}$Ga-FAPI-46 just after purification (1µL injection) shows a >99% RCP (**A**). After 40 min at room temperature, the RCP of the product decreased to 93.75% (**B**) (10µL injection). The retention time ($R_t$) is indicated as min:s. Each peak was also expressed as %ROI (region of interest, bottom value).

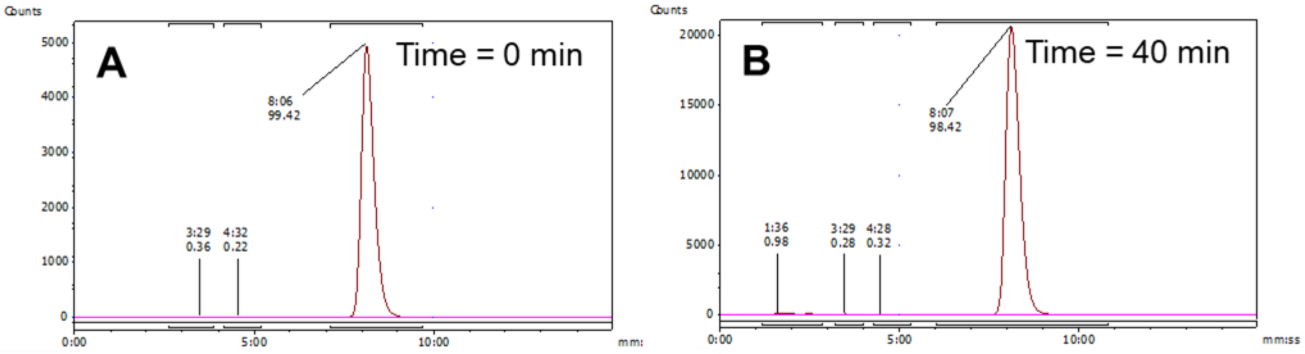


**Figure S6**. Ascorbic acid added to the final product: the radiochromatogram of $^{68}$Ga-FAPI-46 just after purification (1µL injection) shows a >99% RCP (**A**). After 40 min at room temperature, the RCP of the product was still >98% (**B**) (10µL injection). The retention time ($R_t$) is indicated as min:s. Each peak was also expressed as %ROI (region of interest, bottom value).

**ITLC analysis of product**

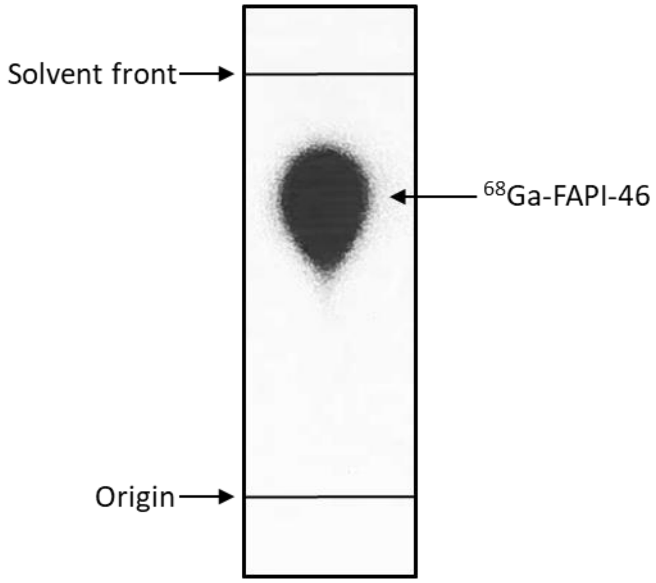


**Figure S7**. Instant thin layer chromatography analysis (ITLC) of purified $^{68}$Ga-FAPI-46 using ITLC-SG strips and 75% methanol 25% 5 M ammonium acetate pH 7 as mobile phase. The product has an $R_f$ of 0.8. Any free $^{68}$Ga would be present at the origin as insoluble $^{68}Ga(OH)_3$.

**PET imaging**

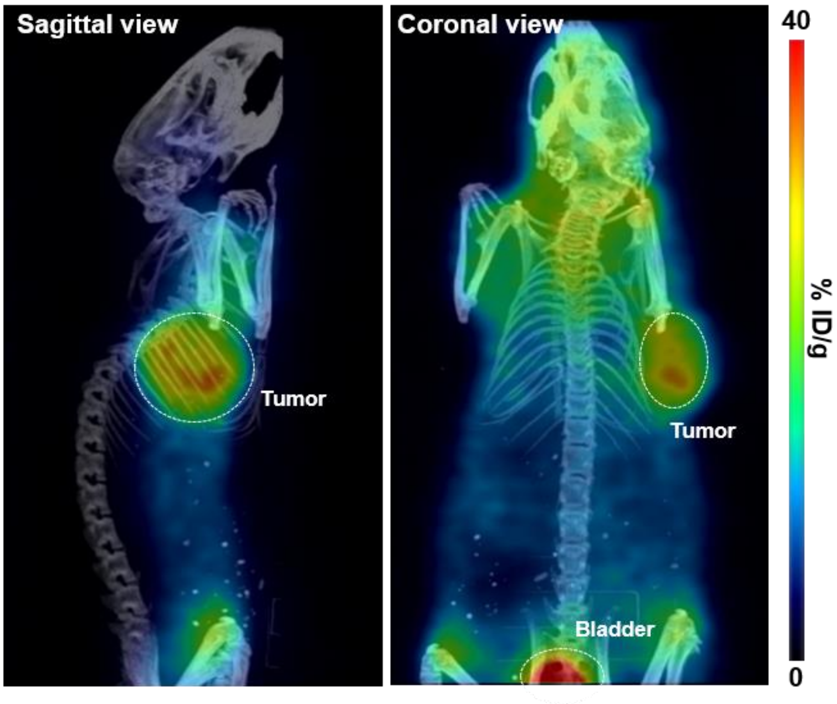


**Figure S8**. Representative PET/CT image of $^{68}$Ga-FAPI-46 in a mouse bearing subcutaneous U87-MGvIII tumor. Tumor and bladder are outlined by a white circle. As confirmed by the biodistribution data (Table S1), there is a clear accumulation of radioactivity in non-target organs such as bone that result in a high background signal. The high bone uptake is possibly caused by the high FAP expression in the bone marrow.

## Biodistribution table

| Organ | $^{68}$Ga-FAPI-46 uptake (%ID/g) |
|---|---|
| Blood | 2.28 ± 0.36 |
| Heart | 0.99 ± 0.11 |
| Lungs | 1.07 ± 0.05 |
| Liver | 0.56 ± 0.06 |
| Spleen | 0.51 ± 0.04 |
| Stomach | 0.81 ± 0.18 |
| Pancreas | 1.51 ± 0.57 |
| Large Intestine | 0.86 ± 0.27 |
| Kidney | 2.45 ± 0.94 |
| Muscle | 1.77 ± 0.67 |
| Bone | 3.83 ± 1.19 |
| Tumor | 6.41 ± 1.11 |

**Table S1**. Biodistribution results for $^{68}$Ga-FAPI-46 (2 MBq/mouse) at 45 min p.i. The data are reported as the mean percentage of the injected dose per gram of tissue (%ID/g) ± SD (for each group, n =3).
